# UBE2E3 regulates cellular senescence and osteogenic differentiation of BMSCs during aging

**DOI:** 10.7717/peerj.12253

**Published:** 2021-11-18

**Authors:** Yalin Liu, Guangping Cai, Peng Chen, Tiejian Jiang, Zhuying Xia

**Affiliations:** 1 Department of Endocrinology, Endocrinology Research Center, Xiangya Hospital of Central South University, Changsha, China; 2 Department of Orthopedic, Xiangya Hospital of Central South University, Changsha, China

**Keywords:** UBE2E3, Osteoporosis, Osteogenic differentiation, Senescence, Nrf2

## Abstract

**Background:**

Osteoporosis has gradually become a public health problem in the world. However, the exact molecular mechanism of osteoporosis still remains unclear. Senescence and osteogenic differentiation inhibition of bone marrow mesenchymal stem cells (BMSCs ) are supposed to play an important part in osteoporosis.

**Methods:**

We used two gene expression profiles (GSE35956 and GSE35958) associated with osteoporosis and selected the promising gene Ubiquitin-conjugating enzyme E2 E3 (UBE2E3). We then verified its function and mechanism by *in vitro* experiments.

**Results:**

UBE2E3 was highly expressed in the bone marrow and positively associated with osteogenesis related genes. Besides, UBE2E3 expression reduced in old BMSCs compared with that in young BMSCs. In *in vitro* experiments, knockdown of UBE2E3 accelerated cellular senescence and inhibited osteogenic differentiation of young BMSCs. On the other hand, overexpression of UBE2E3 attenuated cellular senescence as well as enhanced osteogenic differentiation of old BMSCs. Mechanistically, UBE2E3 might regulate the nuclear factor erythroid 2-related factor (Nrf2) and control its function, thus affecting the senescence and osteogenic differentiation of BMSCs.

**Conclusion:**

UBE2E3 may be potentially involved in the pathogenesis of osteoporosis by regulating cellular senescence and osteogenic differentiation of BMSCs.

## Introduction

Currently, osteoporosis is a major public health problem in the world, with more than 200 million people suffering from osteoporosis. The incidence of osteoporosis increases with age, affecting more than 70% of the elderly population who are 80 years old and over. There are approximately nine million fractures caused by osteoporosis each year (Prince et al., 2019). Osteoporosis features low bone density and destruction of bone microstructure (Chevalier et al., 2020), ultimately leading to easier fractures (Compston, McClung & Leslie, 2019; Sinnott-Armstrong et al., 2021). Primary osteoporosis is associated with the aging process and the decline of sex hormones (Compston, McClung & Leslie, 2019; Hayashi et al., 2019). The pathophysiological basis of osteoporosis is the loss of bone quality due to the imbalance of bone formation and resorption (Guo et al., 2021). Bone mass usually peaks in the third decade in most individuals, after which bone resorption takes precedence over bone formation. Accelerated bone loss or failure of achieving normal peak of bone mass may lead to osteoporosis (Varacallo & Fox, 2014). However, the exact molecular mechanism of osteoporosis still remains unclear. It is of great significance for the prevention and treatment of osteoporosis to take a proactive approach to identifying the related factors of primary osteoporosis and predicting high-risk patients in advance (Sanders & Geraci, 2013).

The age-related bone loss usually results from the imbalance of bone remodeling (Rendina-Ruedy & Rosen, 2020), with increased bone resorption and decreased bone formation (Berger et al., 2019; Martin & Sims, 2005; Parfitt, 1982; Yu et al., 2019). BMSCs are undifferentiated cells with multi-differentiation capabilities (Ng, Kuncewicz & Karp, 2015), which are closely related to the progress of osteoporosis (Berebichez-Fridman & Montero-Olvera, 2018; Lu et al., 2016). In recent decades, BMSCs have been widely used in basic research (Guo et al., 2020; Li et al., 2015; Li et al., 2018; Lyu et al., 2020; Su et al., 2019) and have a good application prospect in the treatment of osteoporosis (Kolios & Moodley, 2013; Li et al., 2009; Xie et al., 2014). Studies have also indicated that aging is related to the increase of cellular senescence (Childs et al., 2015), and BMSCs undergo senescence in the process of bone aging (Yang et al., 2017; Zhou et al., 2020).

Based on the development of high-throughput screening technology, gene microarray analysis has become a powerful instrument to identify differentially expressed genes (DEGs), which may suggest potential biomarkers in many diseases (Peng et al., 2020). Gene microarray analysis has been used on the pathogenesis of osteoporosis (Ma et al., 2017). We used bioinformatics technology to explore and verify osteoporosis related hub genes, among which UBE2E3 decreased in old BMSCs compared with that in young BMSCs and was positively associated with osteogenesis related genes. We chose UBE2E3 for further verification and found that UBE2E3 affected the senescence and osteogenic differentiation of BMSCs. More explicitly, knockdown of UBE2E3 significantly promoted cellular senescence in BMSCs, and inhibited the osteogenic differentiation of young BMSCs. On the other hand, overexpression of UBE2E3 attenuated cellular senescence and enhanced osteogenic differentiation of old BMSCs. Mechanistically, UBE2E3 might regulate the nuclear translocation of Nrf2 and its activity, thus affecting the senescence and osteogenic differentiation of BMSCs. Additionally, our finding of potential related DEGs enrichment pathways might be useful in the further study of the exploration of osteoporosis mechanisms.

## Materials & Methods

### GEO gene expression data

Gene Expression Omnibus (GEO) database (https://www.ncbi.nlm.nih.gov/geo/) was created by NCBI, containing numerous gene expression data from research institutions worldwide. We downloaded two gene expression datasets (GSE35956 and GSE35958) from the GEO database. The expression profiling arrays of GSE35956 were generated through the application of GPL570 [HG-U133_Plus_2] Affymetrix Human Genome U133 Plus 2.0 Array, including five samples of human mesenchymal stem cells from osteoporosis patients and the other five bone marrow from non-osteoporotic donors after total hip arthroplasty. Besides, the expression profiling arrays of GSE35958 were also generated through the application of GPL570 [HG-U133_Plus_2] Affymetrix Human Genome U133 Plus 2.0 Array, including five osteoporosis samples and four control samples.

### Data processing

The original CEL files downloaded from GEO were normalized by R software using R package of “limma” (Ritchie et al., 2015). Based on the platform annotation file, the probe ID was then replaced by the corresponding gene symbol. After calculating the missing value by the KNN method, we used the limma R package to screen each data set for DEGs, by limiting the —log2fold change (FC)— > 2 and *p*-value < 0.01. The volcano plots and heatmaps of DEGs were generated afterwards. Finally, we intersected DEGs from the two data sets by using Venn diagrams (http://bioinformatics.psb.ugent.be/webtools/Venn/) for subsequent analysis.

### GO and KEGG pathway analysis of DEGs

We used the “clusterProfiler” package (Yu et al., 2012) in R to analyze and visualize the function of genomic coordinates. Gene ontology (GO) term analysis is a method which contains three parts: biological process (BP), cell component (CC), and molecular function (MF), while Kyoto Encyclopedia of Genes and Genomes (KEGG) pathway is a bioinformatics Database which analyzes gene functions and enriched pathways. The GO term and the KEGG pathway analyses were performed for DEGs with the *p*-value and the *q*-value cutoff set to 0.05.

### Protein–protein interaction (PPI) network construction and hub gene identification

We predicted the PPI network from the shared DEGs using the online Search Tool for the Retrieval of Interacting Genes (STRING) database (http://string-db.org/),which was designed for protein–protein interaction network analyses. Moreover, Cytoscape was used to visualize the constructed PPI network. The top3 most important modules were obtained on Molecular Complex Detection (MCODE) app with the criteria degree cut-off = 2, node score cut-off = 0.2, Max depth = 100, and k-score = 2. In the end, we selected hub genes according to the top 10 nodes ranked by degree through the use of cytoHubba.

### Expression and co-expression analysis of UBE2E3

The expression of UBE2E3 was downloaded from the Genotype Tissue Expression (GTEx) (https://www.gtexportal.org/) and analyzed in different normal tissues. Furthermore, several osteogenesis related genes including RUNX2, COL1A1, BMP2, FOXP1, ALPL, BGLAP and TAZ were also downloaded. We then calculated the Pearson correlation (r) and *p*-value in R, and analyzed the expression of UBE2E3 in the single-cell sequencing data between young and old rat (Ma et al., 2020).

### Animal

The animal study was conducted under the approval of Xiangya Hospital (Central South University) Ethics Committee. Xiangya Hospital of Central South University Ethics Committee approved this research (201703272). C57BL/6 mice were obtained from the Department of laboratory Animals of Central South University, and kept under the specific-pathogen-free (SPF) (2-month mice, *N* = 12; 15-month mice, *N* = 8). They were housed under controlled temperature, and with adequate food and water and were only used to isolate BMSCs. All animals were euthanized to obtain specimens, and we followed the AVMA guidelines in the procedures of euthanizing animals.

### Cell culture and transfection

BMSCs were flushed out from tibia and femur of 2-month-old (young BMSCs) and 15-month-old (old BMSCs) mice and cultured in α-MEM with 10% fetal bovine serum (Gibco), 100 units/ml penicillin and 100 µg/ml streptomycin at 37 °C, 5% CO_2_ humidity environment. The siUBE2E3 and siNC (Ribibio, Guangzhou, China) were transfected into young BMSCs (from 2-month mice) through the use of lipofectamine RNAiMAX (Invitrogen) according to manufacturer’s instructions. Besides, mUBE2E3 pcDNA3.1-HA-C (Youbio Biological Technology Co., Ltd) were also transfected into old BMSCs (from 15-month mice) with lipofectamine 2000 (Invitrogen) to induce UBE2E3 expression.

### qRT-PCR analysis

Trizol reagent (Accurate Biotechnology) was used to extract total RNA from cultured cells for mRNA expression analysis. The reverse transcription kit (Accurate Biotechnology) was used to reverse transcribe 1 µg RNA into first strand cDNA. Finally, we used SYBR Green PCR Master Mix (Accurate Biotechnology) to perform qRT-PCR. Primer sequences can be seen in Table S1.

### Western blot

Western blot analysis was used to measure UBE2E3 expression. Firstly, total cell lysates were separated by sodium dodecyl sulfate (SDS)-polyacrylamide gel electrophoresis (PAGE) and transferred to polyvinylidene fluoride (PVDF) membranes. Next, membranes were blocked at room temperature with 5% defatted milk for 1 h. Then the membrane was incubated with anti- UBE2E3 (bs-8352R, 1:500; Bioss), anti- GAPDH (10494-1-AP,1:5000; Proteintech) overnight at 4 °C. After being washed three times with tris-buffer saline and Tween (TBST) for 10 min each, the membranes were incubated with corresponding horseradish peroxidase-conjugated second antibodies. Immunoreactivity was detected by enhancing chemiluminescence reaction.

### SA-β-gal staining and observation of senescent cells

Cellular senescence-associated β-galactosidase (SA-β-gal) staining was conducted according to the instructions of the β-gal staining kit (Solarbio Science & Technology). Briefly, after PBS wash, BMSCs were fixed with 4% paraformaldehyde for 15 min, and incubated overnight at 37 °C with staining solution.

### Osteogenic differentiation assay

BMSCs were cultured in 12-well plates at 2.5×10^5^ cells/well with osteogenic differentiation media containing 10mM β-glycerol phosphate, 0.1uM dexamethasone, and 50 µM ascorbate-2-phosphate. Alkaline phosphatase (ALP) staining, ALP activity detection (Beyotime Biotechnology) and Alizarin Red staining (ARS) (Cyagen Biosciences Inc) were conducted according to manufacturer’s instructions.

### Immunofluorescence

Immunofluorescence was conducted as previous reported instructions (Plafker & Plafker, 2015). Firstly, cells were fixed with 4% paraformaldehyde at room temperature for 15 min. Then the cells were blocked with 5% Bovine Serum Albumin (BSA) for 60 min. After aspirating the blocking buffers, we added the diluted primary antibody (Cell Signaling Technology, 12721, 1:200) to incubate at 4 °C overnight. Then the cells were incubated with Alexa488 nm-conjugated secondary antibodies. Finally, the nucleus was stained with Dapi.

### Statistical analysis

All data were displayed as mean ± SD. SPSS 22.0 was used for statistical analysis. Two-tailed Student’s t-tests were conducted for two group comparisons. Statistical significance was considered at *P* < 0.05 (∗*P* < 0.05, ∗ ∗P <0.01, ∗ ∗∗*P* < 0.001).

## Results

### Identification of DEGS in osteoporosis

**Figure 1:**
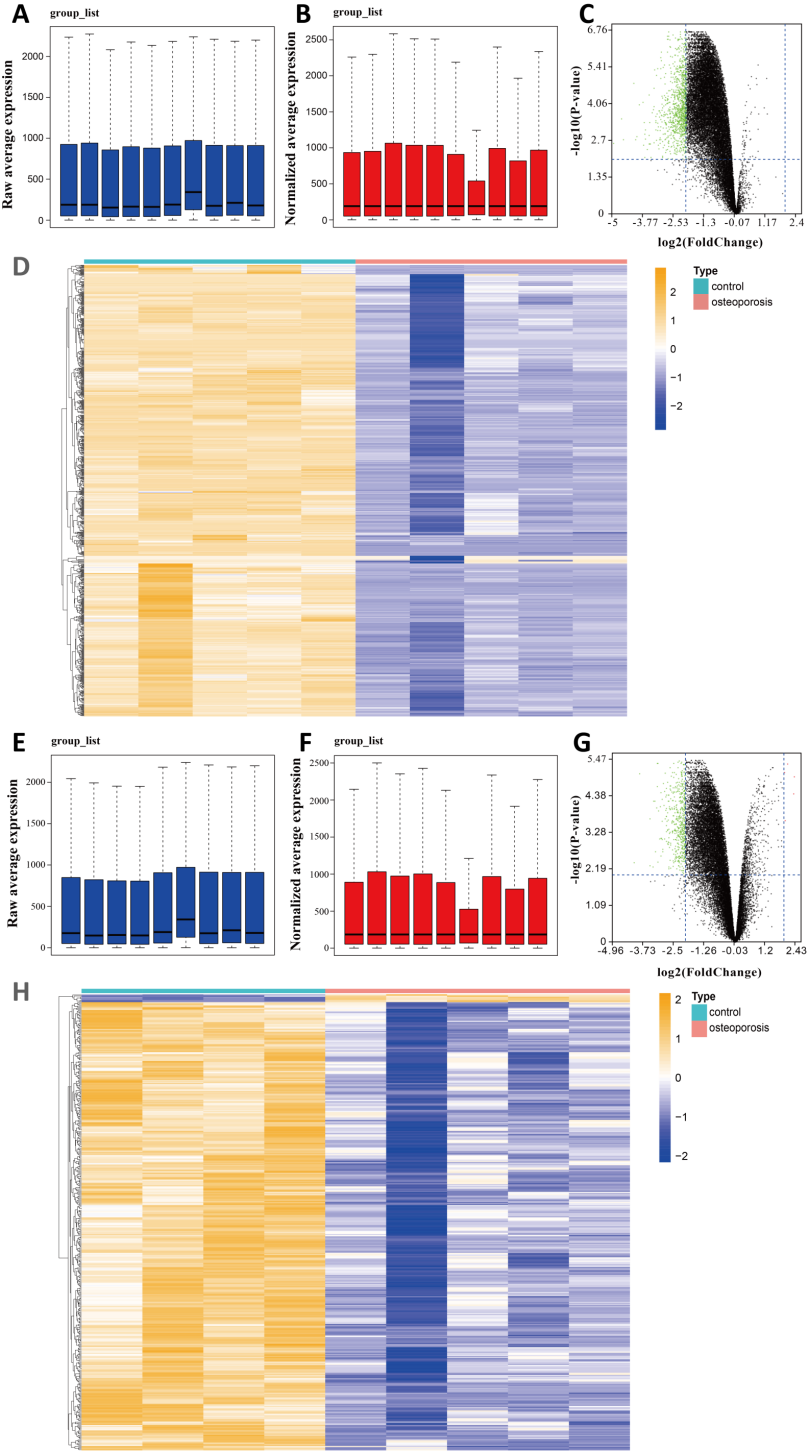
Identifying DEGs in BMSCs from osteoporosis patients and non-osteoporotic donors (GSE35956 and GSE35958). (A) Boxplot of raw mRNA expression in GSE35956. (B) Boxplot of normalized mRNA expression in GSE35956. (C) Volcano Plot of all normalized mRNA expression in GSE35956. (D) Heatmap of DEGs in BMSCs from GSE35956. —log FC— ≥ 2, *P* value < 0.01. (E) Boxplot of raw mRNA expression in GSE35958. (F) Boxplot of normalized mRNA expression in GSE35958. (G) Volcano Plot of all normalized mRNA expression in GSE35958. (H) Heatmap of DEGs in BMSCs from GSE35958. —log FC— ≥ 2, *P* value < 0.01.

We selected two gene expression profiles (GSE35956 and GSE35958) associated with osteoporosis from the GEO database. After normalizing the profiles, we first conducted gene differential analysis by comparing bone marrow of osteoporosis patients and non-osteoporotic donors (Figs. 1A, 1B, 1E, 1F). By limiting —log FC— ≥ 2 and *P* value < 0.01, a total of 883 downregulated DEGs were identified from GSE35956. In another GSE35958 database, 411 DEGs were identified, including seven genes upregulated and 404 genes downregulated. The volcano plots and heatmaps showed the differential expression of these genes from GSE35956 (Figs. 1C, 1D) and GSE35958 (Figs. 1G, 1H) separately. A total of 176 shared DEGs intersected by the two DEGs sets were then obtained. In general, 176 downregulated DEGs were considered to be associated with osteoporosis for subsequent analysis, while no DEGs were upregulated.

### Functional enrichment analyses of DEGs

**Figure 2:**
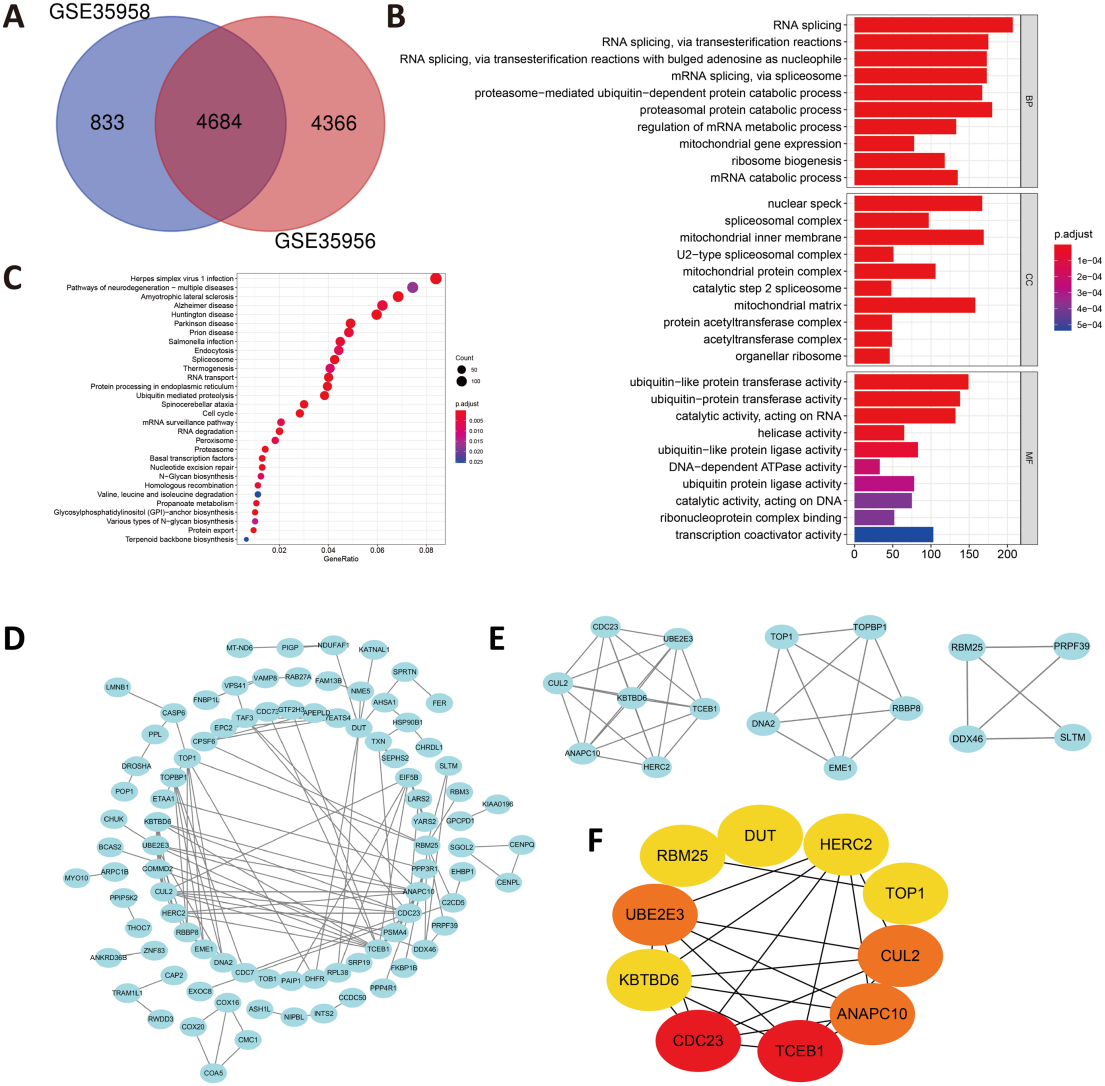
(A) An extended range of DEGs were selected from GSE35956 and GSE35958. —log FC— ≥ 1, *P* value < 0.05. (B) GO analysis of DEGs. (C) KEGG analysis of DEGs. (D) The PPI network of DEGs and downregulated genes were marked in blue. (E) Top three most important modules were obtained from PPI network. (F) HUB genes were selected according to the top10 nodes ranked by degree using cytoHubba.

In view of the small number of DEGs selected above, hardly can significant enrichment results be obtained. In that case, we have appropriately relaxed the limitation of the selection of DEGs as —log FC— ≥ 1 and *P* value < 0.05. After intersecting the separate DEGs from GSE35956 and GSE35958, we obtained 4,684 DEGs to conduct biological annotation (Fig. 2A). The GO term enrichment analysis results were composed of: BP, CC, and MF (Fig. 2B). The BP result showed significant enrichment in RNA splicing, proteasomal protein catabolic process and RNA splicing, *via* transesterification reactions. In the CC group, the DEGs were mainly enriched in nuclear speck, spliceosomal complex as well as mitochondrial inner membrane. Moreover, as for the MF, the DEGs were enriched in ubiquitin-like protein transferase activity, catalytic activity, acting on RNA, helicase activity and so on. We also performed the KEGG pathways analysis which identified that the downregulated DEGs were mainly enriched in herpes simplex virus 1 infection, pathways of neurodegeneration-multiple diseases, amyotrophic lateral sclerosis, and Alzheimer disease (Fig. 2C).

### Construction of the PPI network and identification of hub genes

In order to predict the interactive relationships among DEGs, we constructed the PPI Network of the 176 DEGs by using the STRING database. After screening, we included a total of 89 nodes and 131 edges in the PPI Network, which was presented by the Cytoscape software (Fig. 2D). Moreover, we then obtained the top three most important modules by using MCODE app in Cytoscape (Fig. 2E). We selected ElonginC (TCEB1), Cell Division Cycle 23 (CDC23), UBE2E3, Cullin-2 (CUL2), Anaphase-promoting complex subunit 10 (ANAPC10), Deoxyuridine 5′-triphosphate nucleotidohydrolase, mitochondrial (DUT), E3 ubiquitin-protein ligase (HERC2), Kelch repeat and BTB domain-containing protein 6 (KBTBD6), DNA topoisomerase 1 alpha (TOP1) and RNA-binding protein 25 (RBM25) as hub genes according to the top10 nodes ranked by degree by using cytoHubba (Fig. 2F). Among these 10 hub genes, we selected UBE2E3 with the following properties: (a) UBE2E3 was highly expressed in the bone marrow; (b) UBE2E3 was positively associated with osteogenesis related genes; (c) UBE2E3 expression reduced in old BMSCs compared with that in young BMSCs; (d) UBE2E3 is a kind of highly conserved metazoan enzyme that pairs with E3 ligases to conjugate Mono-Ub on substrates (Nguyen et al., 2014); and (e) Studies have shown that UBE2E3 has an close relationship with cellular senescence (Plafker et al., 2008; Plafker et al., 2018). Since osteoporosis has a close relationship with age, we selected UBE2E3 as the promising gene that might be associated with osteoporosis.

### Validation of UBE2E3 expression

**Figure 3:**
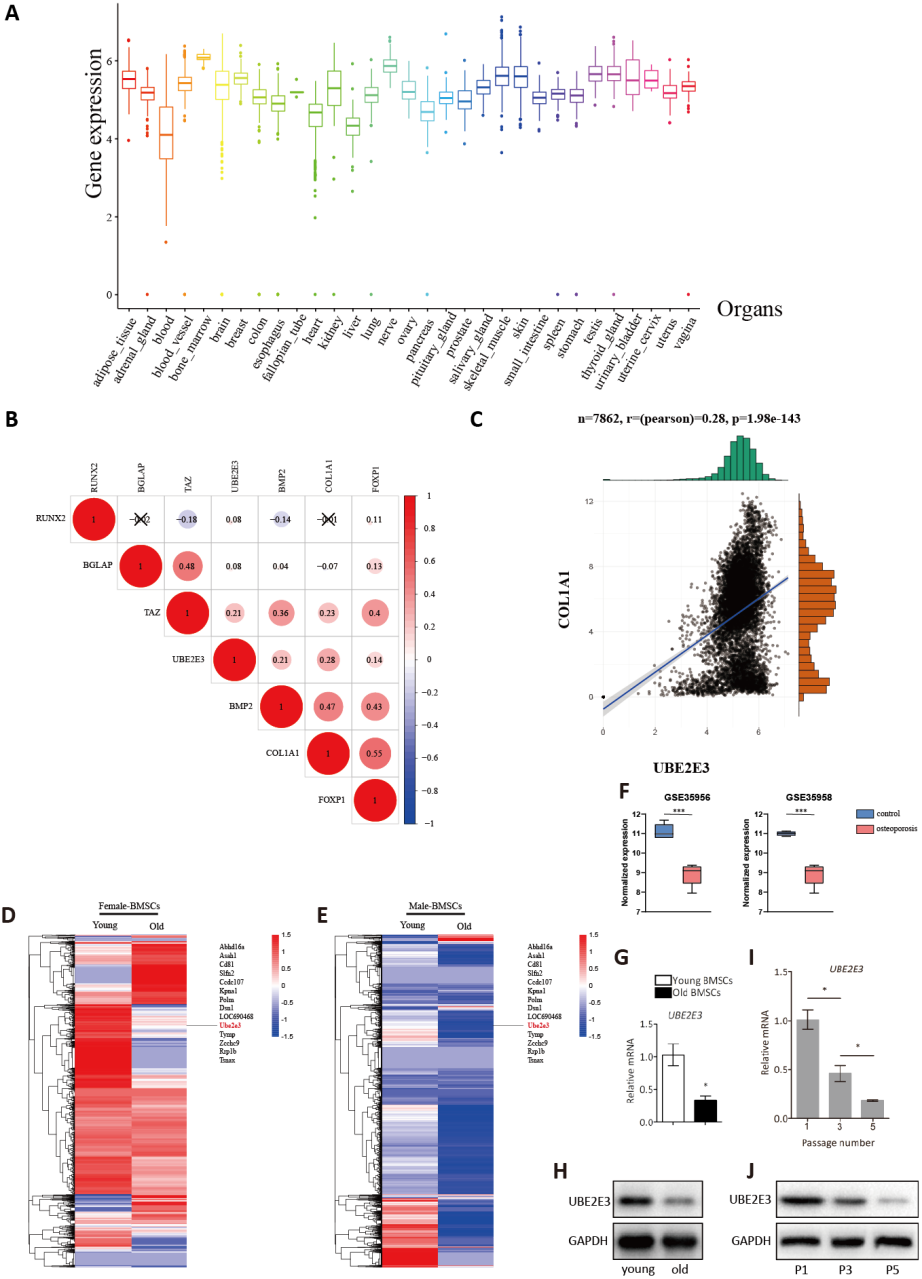
Validation of UBE2E3 expression. (A) The expression of UBE2E3 in 7862 normal tissues of GTEx. (B) The correlation analysis of UBE2E3 with osteogenesis related genes in GTEx. (C) The correlation analysis of COL1A1 and UBE2E3 in GTEx. (D, E) Heatmap of single cell sequencing of rat BMSCs. (F) UBE2E3 expression of human BMSCs from GSE35956 and GSE35958. (G) qRT-PCR analyses of UBE2E3 levels in old mouse BMSCs and young mouse BMSCs. (H) Western blot analyses of UBE2E3 levels in old mouse BMSCs and young mouse BMSCs. (I) qRT-PCR analyses of UBE2E3 levels in increasing numbers of passages of mice BMSCs. (J) Western blot analyses of UBE2E3 levels in increasing numbers of passages of mice BMSCs. Error bars showed standard deviation. *P < 0.05, ***P < 0.001.

To investigate whether UBE2E3 plays a part in bone formation, we explored the expression of UBE2E3 in 7,862 normal tissues of GTEx. Among the 30 tissues, we found that UBE2E3 was highly expressed in the bone marrow (Fig. 3A). Furthermore, we analyzed the co-expression of UBE2E3 with osteogenic related genes including RUNX2, COL1A1, BMP2, FOXP1, ALPL, BGLAP and TAZ in normal tissues from GTEx (Fig. 3B). The correlations of UBE2E3 with all the selected osteogenesis related genes were positive, suggesting that UBE2E3 may have a close relationship with bone formation. COL1A1 had the largest coefficient of association with UBE2E3 (Fig. 3C). Besides, analysis of single cell sequencing of rat BMSCs showed that UBE2E3 expression was significantly lower in BMSCs from old rats compared with that from younger ones (Figs. 3D, 3E) (Ma et al., 2020). In addition, we found that UBE2E3 significantly decreased in BMSCs of patients with osteoporosis from GSE35956 and GSE35958 (Fig. 3F). Similarly, qRT-PCR and western blot analyses showed that UBE2E3 level was significantly lower in BMSCs from old mice compared with that from young mice, which indicated its lower expression with the development of age (Figs. 3G, 3H). Of note, as the number of passages increased, the expression of UBE2E3 also gradually decreased in mice BMSCs (Figs. 3I, 3J).

### UBE2E3 regulates senescence and osteogenic differentiation of BMSCs

**Figure 4:**
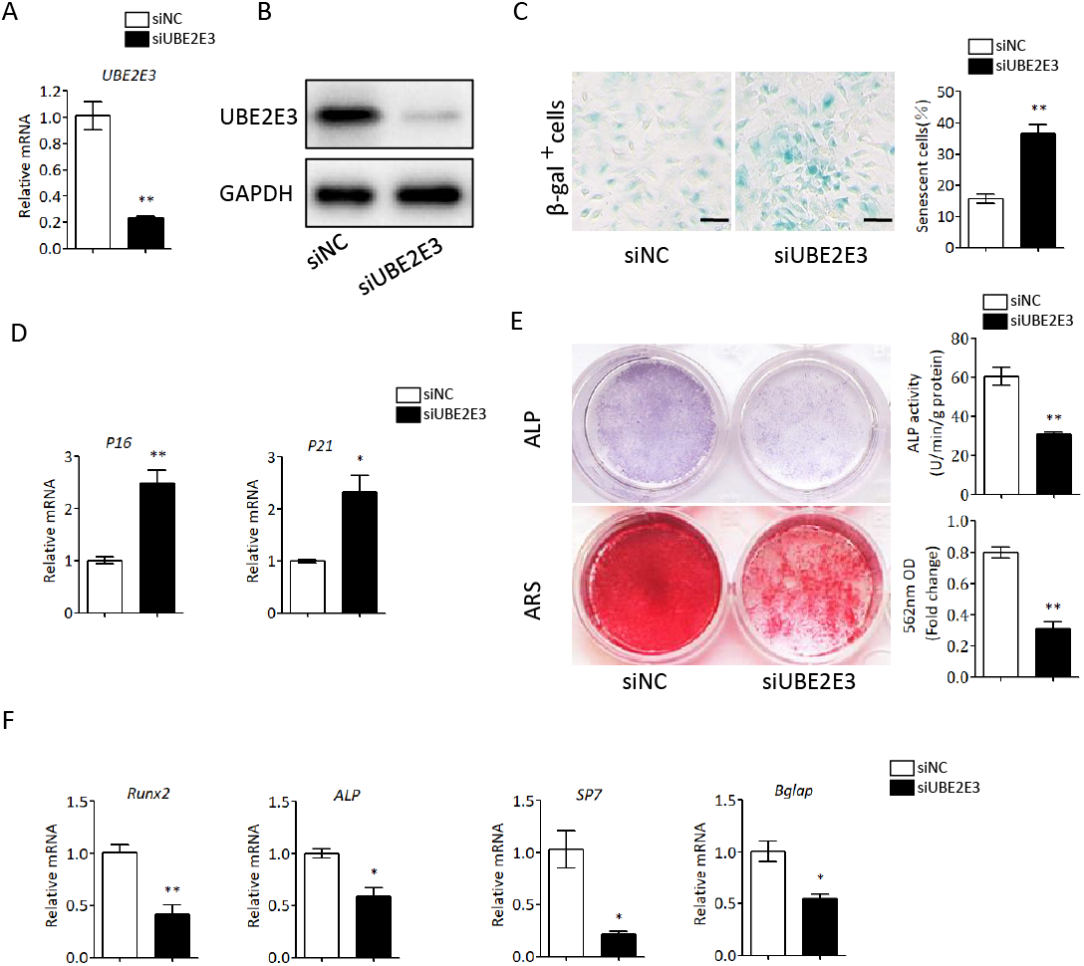
Knockdown of UBE2E3 accelerated cell senescence and inhibited osteogenic differentiation of young BMSCs. (A) Knockdown efficiency of UBE2E3 was verified by qRT-PCR. (B) Knockdown efficiency of UBE2E3 was verified by western blot. (C) β-Gal staining and quantification of senescent BMSCs in siUBE2E3 group and siNC group. (D) The relative mRNA expression of p16 and p21 between siUBE2E3 group and siNC group. (E) ALP and ARS staining results in siUBE2E3 group and siNC group, with the quantitative analysis. (F) The relative mRNA expression of RUN2, ALP, SP7 and BGLAP between siUBE2E3 group and siNC group. Error bars showed standard deviation. *P < 0.05, **P < 0.01.

We first flushed out and cultured BMSCs from bone marrow of 2-month-old mice, and further knocked down UBE2E3 with siRNA, whose effect was verified by qRT-PCR and western blot (Figs. 4A, 4B). When verifying the effect of UBE2E3 on senescence of BMSCs, we found that 6 days post-siRNA administration, the number of SA-β-gal positive cells in UBE2E3 knockdown group increased significantly compared with the control group (Fig. 4C), and the transcription level of p16 (Omori et al., 2020; Xiao et al., 2020) and p21 (Zhang et al., 2019), markers of cellular senescence, also increased (Fig. 4D). In order to explore the effect of UBE2E3 on the osteogenic differentiation of BMSCs, we induced osteogenic differentiation of BMSCs *in vitro*. The results showed that knockdown of UBE2E3 significantly inhibited the osteogenic differentiation of young BMSCs as analyzed by ALP staining and ARS results (Fig. 4E). Furthermore, the mRNA levels of the osteogenic differentiation related genes, RUN2, ALP, SP7 and BGLAP, decreased with UBE2E3 knockdown (Fig. 4F).

**Figure 5:**
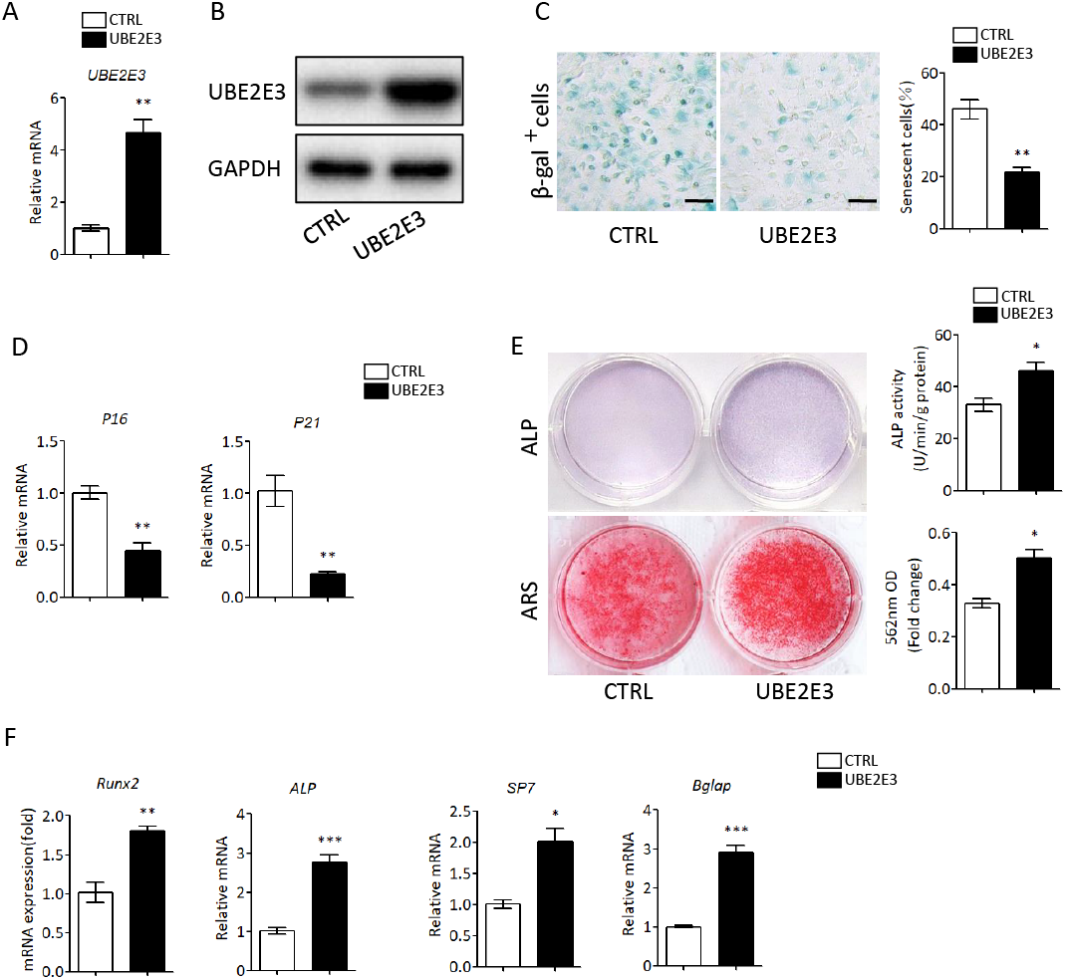
Overexpression of UBE2E3 attenuated cell senescence as well as enhancing osteogenic differentiation of old BMSCs. (A) Overexpression efficiency of UBE2E3 was verified by qRT-PCR. (B) Overexpression efficiency of UBE2E3 was verified by western blot. (C) β-Gal staining and quantification of senescent BMSCs in UBE2E3 overexpression group and the control group. (D) The relative mRNA expression of p16 and p21 between UBE2E3 overexpression group and the control group. (E) ALP and ARS staining results in UBE2E3 overexpression group and the control group, with the quantitative analysis. (F) The relative mRNA expression of RUN2, ALP, SP7 and BGLAP between UBE2E3 overexpression group and the control group. Error bars showed standard deviation. *P < 0.05, **P < 0.01, ***P < 0.001.

Likewise, we overexpressed UBE2E3 in old BMSCs with plasmid, which was verified by qRT-PCR and western blot (Figs. 5A, 5B). We found that the number of SA-β-gal positive cells in UBE2E3 overexpression group decreased significantly (Fig. 5C), and the transcription level of p16 and p21 also reduced (Fig. 5D). On the other hand, the upregulation of UBE2E3 significantly promoted the osteogenic differentiation of old BMSCs as analyzed by ALP and ARS staining (Fig. 5E). Additionally, the mRNA levels of osteogenic differentiation related genes increased in UBE2E3 overexpression group (Fig. 5F). Collectively, UBE2E3 regulated the senescence and osteogenic differentiation of BMSCs.

### UBE2E3 regulates cellular senescence and osteogenic differentiation through controlling Nrf2 distribution and activity

**Figure 6:**
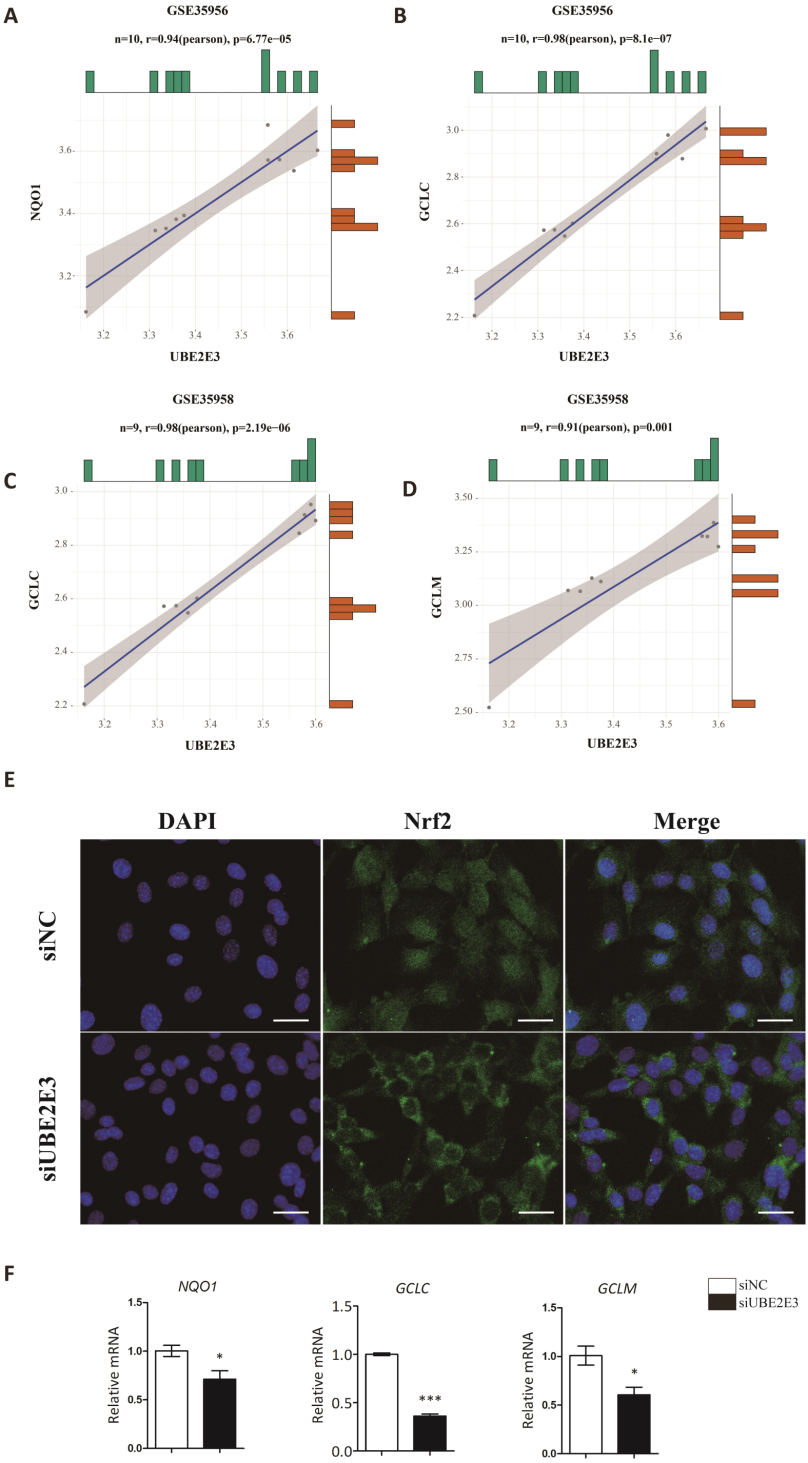
UBE2E3 regulates BMSCs senescence and osteogenic differentiation by controlling Nrf2 distribution and activity. (A) The correlation of NQO1 and UBE2E3 in GSE35956. (B) The correlation analysis of GCLC and UBE2E3 in GSE35956. (C) The correlation analysis of GCLC and UBE2E3 in GSE35958. (D) The correlation of GCLM and UBE2E3 in GSE35958. (E) Representative images of BMSCs treated with siNC and siUBE2E3, and stained with DAPI and antibody against Nrf2. Scale bar, 50 µm. (F) NQO1, GCLC and GCLM expression between siUBE2E3 group and siNC group as analyzed by qRT-PCR. Error bars showed standard deviation. *P < 0.05, ***P < 0.001.

**Figure 7:**
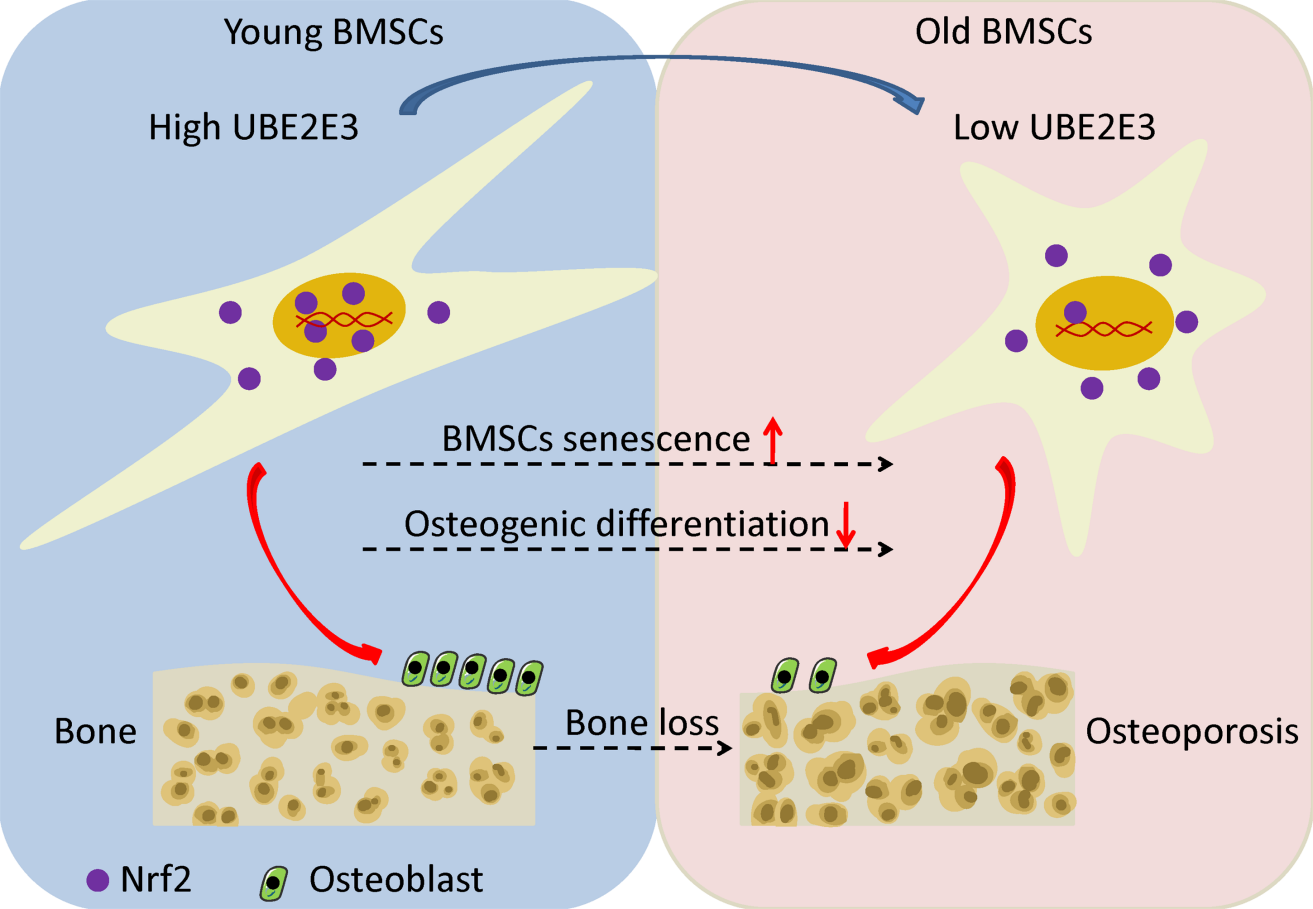
The schematic of UBE2E3 regulating senescence and osteogenic differentiation of BMSCs during aging. High expression of UBE2E3 contributes to nuclear translation of Nrf2 and its high activity. With age, the expression of UBE2E3 decreases and nuclear accumulation of Nrf2 and its activity are restrained, leading to cell senescence and reduced osteogenic differentiation potential of BMSCs.

Kendra et al. found that UBE2E3 could regulate the nuclear translocation and activity of Nrf2 (Plafker & Plafker, 2015). Moreover, studies showed that Nrf2 played an important role in regulating cellular senescence and osteogenic differentiation (Fei et al., 2021; Jie et al., 2020; Yoon, Choi & Lee, 2016; Yuan et al., 2021). Hence, we examined the correlation of UBE2E3 and Nrf2 downstream genes: NQO1, GCLC, GCLM in GTEx, and interestingly found they are positively correlated (Figs. 6A–6D). In order to verify whether UBE2E3 affects Nrf2 nuclear translocation in BMSCs, we used immunofluorescence to observe the cellular distribution of Nrf2 between the control group and the siUBE2E3 group. We found that in siUBE2E3 cells, the nuclear NFR2 was significantly reduced (Fig. 6E). Furthermore, we found that the transcription of Nrf2 downstream target genes: GCLC, GCLC and NQO1 reduced 3 days after knockdown of UBE2E3, which suggested the reduced Nrf2 activity (Fig. 6F). In general, these results suggested that UBE2E3 might regulate cellular senescence and osteogenic differentiation of BMSCs through controlling Nrf2 distribution and activity (Fig. 7).

## Discussion

Osteoporosis, a kind of metabolic disease, is characterized by a decrease in bone mass in spite of the fact that the ratio of calcium salt to matrix is normal (Golob & Laya, 2015). Generally speaking, 12% of men and 30% of women will be affected by this disease at some point in their lives (Armas & Recker, 2012). As one of the most common chronic diseases of the elderly, osteoporosis usually comes with bone pain and greater possibility of fractures (Golob & Laya, 2015). It has been a serious problem for the world’s public health care system (Rizzoli et al., 2014).

Osteoporosis is a multifactorial disease (De Lima et al., 2019; Tu et al., 2019; Zou et al., 2020), and multiple genes and signal pathways are involved in its pathogenesis (Andreev et al., 2020; Gogakos et al., 2009; Guo et al., 2020; Nishikawa et al., 2015; Wang et al., 2020; Yang et al., 2019; Yu et al., 2021). We selected 176 downregulated DEGs from two gene expression profiles (GSE35956 and GSE35958) associated with osteoporosis and then conducted GO and KEGG analysis of DEGs, which identified the possible enrichment of osteoporosis related genes. Consistent with our enrichment analysis results, RNA splicing has been identified being associated with musculoskeletal disorders (Rai et al., 2021; Wiley et al., 2021; Zhang et al., 2021), and multiple RNA splicing related genes suggest the occurrence of osteoporosis (Liu et al., 2020). Moreover, we found that the downregulated DEGs were enriched in amyotrophic lateral sclerosis and Alzheimer’s disease by KEGG pathways analysis. Accumulation researches hypothesize that neurodegenerative disorders are associated with osteoporosis. Bone mineral density is affected by many factors that influence the balance between osteoblasts and osteoclasts. Several neurotoxic metals have a negative effect on bone density (Aaseth, Boivin & Andersen, 2012; Chen et al., 2014; Li et al., 2011; Wu et al., 2014) and neurodegeneration markers are also detected in bone tissues (Roos, 2014). For example, in both men and women, lower bone density and a higher rate of bone loss are associated with high risk of Alzheimer’s disease, indicating an inherently close relationship between osteoporosis and Alzheimer’s disease (Stern et al., 2021; Zhou et al., 2011). Besides, people with osteoporosis also have an increasing risk of dementia (Chang et al., 2014).

By constructing the PPI network, we selected TCEB1, CDC23, UBE2E3, CUL2, ANAPC10, DUT, HERC2, KBTBD6, TOP1 and RBM25 as hub genes. Consistent with our findings, many studies have reported our selected DEGs with the defect of bone formation. For example, mutations in DUT, a key enzyme preventing the accidental incorporation of uracil to maintain DNA integrity, can lead to a kind of early-onset diabetes accompanied with bone marrow failure (Dos Santos et al., 2017). The expression of HERC2 and SOX18 in osteosarcoma is significantly negatively correlated and SOX18 has been tested to play a significant part in the proliferation, migration, invasion and apoptosis of osteosarcoma cells. Besides, HERC2 interacts with SOX18, which is overexpressed in osteosarcoma cells (Zhu et al., 2018). On the other hand, miR-199a-3p is downregulated in osteosarcoma, and RBM25 may be its potential target gene (Huang et al., 2019).

In our study, UBE2E3 had a high degree score in the PPI network, and it was highly expressed in the bone marrow compared with other normal tissues. Surprisingly, the correlations of UBE2E3 with osteogenic related genes were positive in GTEx and the analysis of single cell sequencing of rat BMSCs (Ma et al., 2020) showed that UBE2E3 expression was significantly lower in older rats compared with that in younger ones. Thus, we finally chose UBE2E3 as a promising gene for further verification. *In vitro* experiments, UBE2E3 was verified to regulate senescence and the osteogenic differentiation of BMSCs, which was consistent with our bioinformatics analysis findings.

Previous studies have shown that depletion of UBE2E3 can lead to a redistribution of Nrf2 from nucleus to the cytoplasm (Plafker et al., 2008; Plafker et al., 2010; Plafker & Plafker, 2015). Our findings were in line with the previous studies that knockdown of UBE2E3 reduced Nrf2 in the nucleus as well as reducing the expression of Nrf2 target genes. Because of downregulation of UBE2E3, the transcriptional activity of Nrf2 reduces and eventually drives proliferating cells into senescence (Plafker et al., 2018), which is closely related to bone disease (Kaur & Farr, 2020). Several studies have shown that the level and activity of Nrf2 might decrease with age (Kopacz et al., 2020; Peng et al., 2019) and Nrf2 gene deletion could lead to premature senescence in embryonic fibroblasts (Kapeta, Chondrogianni & Gonos, 2010). Its silencing can lead to increased oxidative stress, decreased biological functions, and accelerated cellular senescence (Wang et al., 2018), while the selective activator of Nrf2 can prevent this process (Fang et al., 2018; Strong et al., 2016; Wang et al., 2017). In particular, Nrf2 can negatively regulate p53 through regulating the expression of sirtuin 1 (Yoon, Choi & Lee, 2016). Plenty of studies have suggested that the induction and activation of Nrf2 can prevent the occurrence of osteoporosis (Meng et al., 2021), while inhibiting of Nrf2 may be an important feature of osteoporosis (Chen et al., 2021). Besides, the activity of Nrf2 is low in old BMSCs and inhibition of Nrf2 activity inhibits the self-renewal and osteogenic differentiation of BMSCs (Yoon, Choi & Lee, 2016). Moreover, Nrf2 is a transcription factor that regulates several cell protection genes (Zhu et al., 2018), whose expression participate in many important biological functions, including oxidative stress response, protein homeostasis, DNA repair and autophagy during cellular senescence (Huang et al., 2019). In a word, we have demonstrated that UBE2E3 might regulate the nuclear translocation of Nrf2 and control its activity in BMSCs. Hence, UBE2E3 may be potentially involved in regulating cellular senescence and osteogenic differentiation of BMSCs through regulating the Nrf2 localization and activity. The limitation of this study is that it would be more convincing to provide more direct evidence to demonstrate that UBE2E3 regulates senescence and osteogenic differentiation of BMSCs by regulating Nrf2 localization and activity. This can be explored in the future studies.

In summary, our findings revealed that UBE2E3 was highly expressed in the bone marrow and was closely related with osteogenic differentiation and senescence of BMSCs. In that case, UBE2E3 may be a significant target gene in the treatment of osteoporosis.

## Conclusions

Based on the bioinformatics analysis and *in vitro* experiments, we identified the promising gene UBE2E3 for osteoporosis, which regulated the senescence and osteogenic differentiation of BMSCs.

## Supplemental Information

10.7717/peerj.12253/supp-1 Supplemental Information 1 Primer sequence for qRT-PCR F: forward primer; R: reverse primer; qRT-PCR: quantitative real-time PCR.

10.7717/peerj.12253/supp-2 Supplemental Information 2 The raw data of plots in Fig. 3–Fig. 6

10.7717/peerj.12253/supp-3 Supplemental Information 3 Author Checklist

## Additional Information and Declarations

### Data Availability

The following information was supplied regarding data availability: The raw data of plots in Figs. 3–6.
