# Supplementary material for: UBE2E3 regulates cellular senescence and osteogenic differentiation of BMSCs during aging
Source: PeerJ. 2021 Nov 18;9:e12253. doi: 10.7717/peerj.12253 (PMC8606162; doi:10.7717/peerj.12253)
Supplement: Supplemental Information 1 — F: forward primer; R: reverse primer; qRT-PCR: quantitative real-time PCR. [file peerj-09-12253-s001.docx]

Supplemental table 1. Primer sequence for qRT-PCR.

| gene | Primer sequence (5’-3’) |
| --- | --- |
| UBE2E3 | F: GACAACTGGAGTCCTGCTTTGAC |
|  | R: ACTGAGTGGCTATGCTTCCGAC |
| P16 | F: ACATCAAGACATCGTGCGATATT |
|  | R: CCAGCGGTACACAAAGACCA |
| P21 | F: CCTGGTGATGTCCGACCTG |
|  | R: CCATGAGCGCATCGCAATC |
| Runx2 | F: GAAATGCCTCCGCTGTTATG |
|  | R: AGGTGAAACTCTTGCCTCGTC |
| ALP | F: CCAACTCTTTTGTGCCAGAGA |
|  | R: GGCTACATTGGTGTTGAGCTTTT |
| Sp7 | F:ATGGCGTCCTCTCTGCTTG |
|  | R:TGAAAGGTCAGCGTATGGCTT |
| Bglap | F: AGGTGAAACTCTTGCCTCGTC |
|  | R: AAGCAGGAGGGCAATAAGGT |
| GCLM | F:TCCTGCTGTGTGATGCCACCAG |
|  | R: GCTTCCTGGAAACTTGCCTCAG |
| NQO1 | F: GCCGAACACAAGAAGCTGGAAG |
|  | R: GGCAAATCCTGCTACGAGCACT |
| GCLC | F:ACACCTGGATGATGCCAACGAG |
|  | R: CCTCCATTGGTCGGAACTCTAC |
| GAPDH | F:TGTGTCCGTCGTGGATCTGA |
|  | R:CCTGCTTCACCACCTTCTTGA |

F: forward primer; R: reverse primer; qRT-PCR: quantitative real-time PCR.
